# Group A Streptococcus Pneumonia in a Previously Healthy Individual: Is It Still a Thing?

**DOI:** 10.7759/cureus.44539

**Published:** 2023-09-01

**Authors:** Sebastian Casillas, Nicholas Briski, Ahsan Salik, Sadat Kasanga, Ayesha Ali, Mageda Al Areqi, Matthew Yotsuya, Abdallah Khashan

**Affiliations:** 1 Internal Medicine, Raritan Bay Medical Center, Perth Amboy, USA

**Keywords:** pneumonia, tuberculosis-like pneumonia, group a streptococcus, streptococcus pyogenes, streptococcus pneumonia

## Abstract

Group A streptococcus (GAS) is known to cause many different kinds of infections, including invasive pneumonia in rare cases. When it is the causative agent, it is associated with a more severe disease course, but it can often be adequately treated if caught early enough. We hereby present the case of a 32-year-old male with no past medical history who presented with fever, hemoptysis, and tachycardia. Laboratory results showed leukocytosis, hyponatremia, mild transaminitis, and elevated creatine kinase. Initial imaging findings and clinical presentation were concerning for tuberculosis (TB) vs. community-acquired pneumonia (CAP), as it yielded a consolidation in the right upper lobe. The patient had no obvious risk factor except for imprisonment two years prior to symptoms onset. Empirical antibiotics and steroids were started. Quantiferon and acid-fast bacteria (AFB) were negative, but sputum and blood cultures were positive for Streptococcus pyogenes, ruling out TB. Antibiotic therapy was narrowed down. The patient responded well to therapy, with subsequent resolution of symptoms. The current body of knowledge regarding respiratory infections caused by GAS is limited by multiple factors, including its relative rarity and the diversity of how it can present, especially in a developed country. Its mimicry characteristics of other clinical entities, such as TB, can be deceiving, which can delay appropriate treatment if it occurs in settings where the diagnostic tools are not readily available. By sharing more cases and atypical presentations of this disease, the clinical presentations of this pathogen can be more fully understood, and it can be more rapidly identified and treated.

## Introduction

Group A Streptococcus (GAS) is a type of bacteria that is known to cause various diseases since it is able to thrive in multiple organ systems. One of these is the respiratory system; although it is known to be normal flora, it can cause upper respiratory tract infections, and, less commonly, lower respiratory tract infections. The relative rarity of significant infections is partly due to antibiotics, such as penicillin, which have decreased the incidence and prevalence of streptococcal pneumonia [1,2]. However, the incidence has not reached zero. Cases of GAS pneumonia are still observed in high-density population areas such as in the military or prisons. Another condition where infections have been observed is in patients with a previous viral infection, such as influenza, who become vulnerable to a secondary infection with GAS [1,3]. It is less common to observe this infection in previously healthy individuals without exposure to sick contacts. In this case report we will present a sporadic infection in a previously healthy patient.

This case report was previously presented as a meeting abstract at the 2023 Hackensack Meridian Health Resident/Fellow Research Day.

## Case presentation

This patient was a 32-year-old African-American male with no pertinent past medical history who presented to the emergency department (ED) with a fever of 101 F, productive cough, and sharp, right-sided back pain of 10/10, which increased with inspiration and lying down on the same side. Vital signs in the ER included a blood pressure of 104/58 mmHg, heart rate of 112 bpm, respiratory rate of 24 breaths per minute, and oxygen saturation (SpO_2_) of 96%. The cough was persistent and started two days prior with reported white sputum, but on the day of the admission, the patient noted that the sputum was bloody and foul-smelling. The patient denied recent travel or being exposed to sick contacts except for his mother who had coronavirus disease 2019 (COVID-19) two weeks prior. He also denied weight loss or intravenous (IV) drug use. Further questioning revealed that he was incarcerated for two years and was released in 2020.

**Figure 1:**
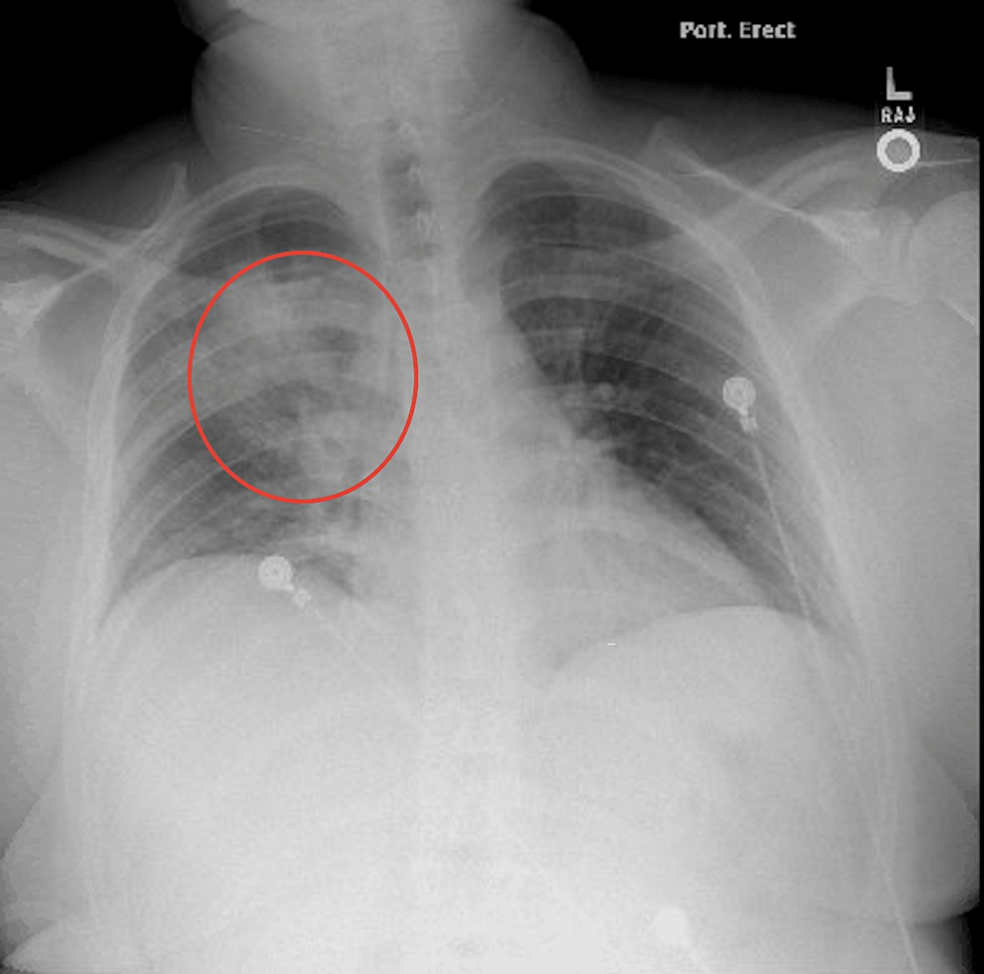
Chest X-ray showing multiple opacities in the right lung

**Figure 2:**
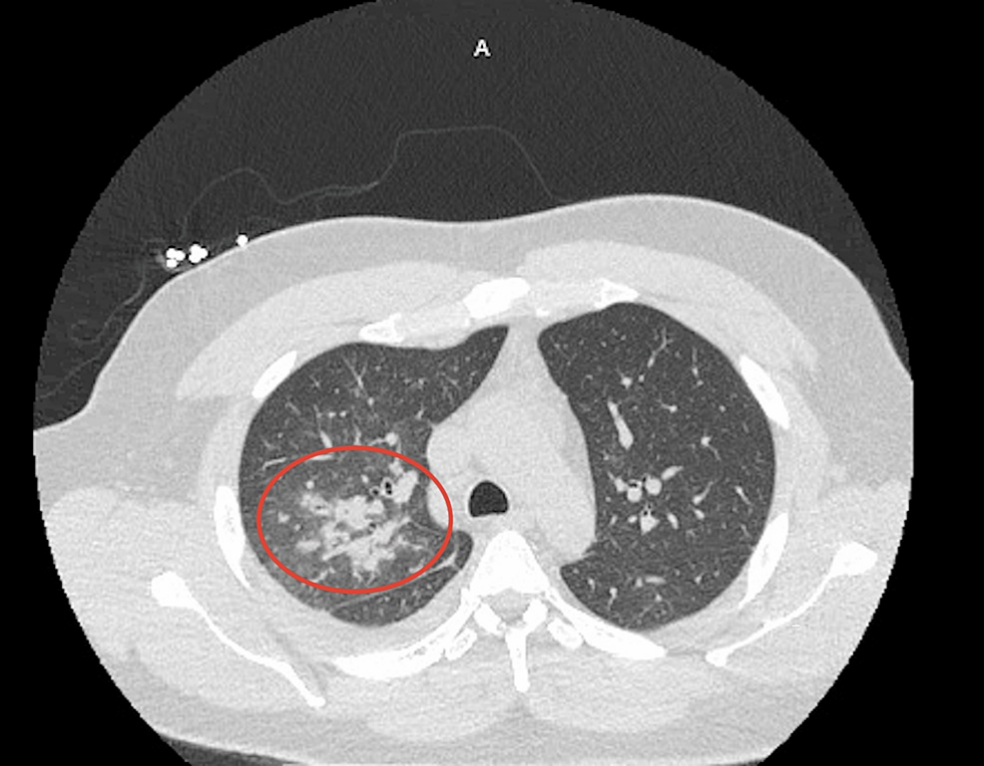
Chest computed tomography (CT) showing multiple nodular lesions in the right lung

**Figure 3:**
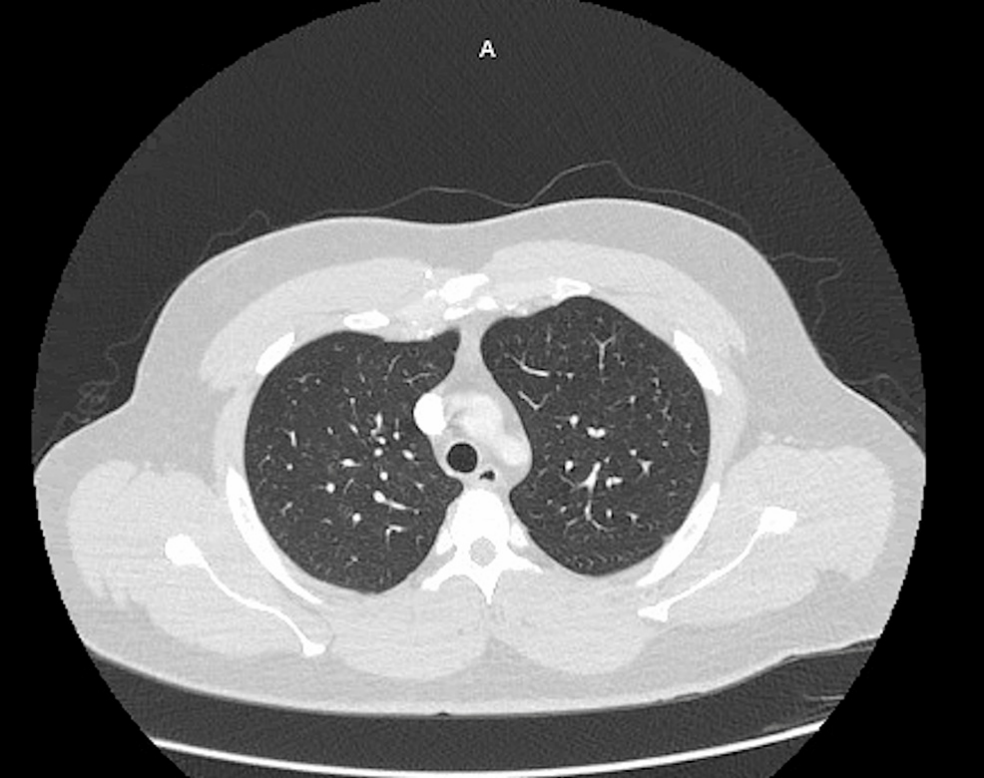
Chest computed tomography (CT) two weeks later, showing resolution of lesions of the right lung

On arrival, the patient was ill-appearing, feverish, and tachycardic. Lung auscultation revealed decreased breath sounds in the right middle and lower fields. Initial workup done by the ED physician consisted of laboratory test and chest X-ray (Figure 1) in addition to empiric ceftriaxone/azithromycin and methylprednisolone, under the suspicion of possible community-acquired pneumonia (CAP) with sepsis, although at this point, tuberculosis (TB) was also suspected. Complete blood count (CBC) results showed an elevated white blood cell count of 21.4 with neutrophilia. Blood chemistry showed hyponatremia of 131, mild transaminitis, and creatinine kinase (CK) of 2849, which raised suspicion for possible rhabdomyolysis. Chest X-ray (CXR) showed a consolidative opacity in the right upper lobe. At this point, infectious disease (ID) was consulted. Initial workup included blood and sputum culture, atypical pneumonia Streptococcus pneumoniae and Legionella urine antigens, acid-fast bacillus (AFB) smear, and culture for TB. The patient was admitted and placed in isolation. The next day, quantiferon testing was ordered as well as two additional AFBs. Ceftriaxone was continued as per ID recommendation. CT of the chest showed multiple, irregular, rounded, mass-like opacities throughout the right lung (Figure 2), predominantly in the upper lobe with surrounding ill-defined ground glass density concerning for TB. After the second day, the WBC started to trend downward, and the CK levels and liver enzymes were normal by day 3. The Streptococcus pneumoniae and Legionella antigens, quantiferon, AFBs, and TB polymerase chain reaction (PCR) testing were all negative. On the third day of hospitalization, the patient began to improve clinically. His hemoptysis stopped as well as fever and malaise, although he reported back pain associated with a persistent cough. At this point, blood and sputum cultures showed moderate growth for beta-hemolytic group A Streptococcus, and the blood culture identification panel identified Streptococcus pyogenes, achieving the final diagnosis. The patient was continued on ceftriaxone to complete a course of 10 days and was discharged on the fifth day. A CT scan done two weeks later (Figure 3) showed significant improvement in the right hemithorax. 

## Discussion

Streptococcus pyogenes is an uncommon but relatively dangerous cause of pneumonia, and the most recent literature shows significant variety in how these cases present. In 2016, Akuzawa and Kurabayashi presented a case review of GAS infections, highlighting that this organism often presents as skin and soft tissue infections, but it was estimated that 10% of GAS cases presented as pneumonia. Additional sources indicate that Group A strep. infections are responsible for less than 1% of community-acquired cases and are usually associated with invasive species such as strains M1 and M3 [3-5]. However, when it manifests as pneumonia, the morbidity caused by this pathogen can vary greatly. There have been cases reported including abscesses, necrotizing pneumonia, cavitations, empyema necessitans, or focal pneumonia. This finding, along with certain risk factors like overcrowding and symptoms like hemoptysis and fever, has the potential to mimic other clinical entities like TB. That being said, these cases can occur in any age demographic and are not disproportionately associated with specific risk factors [6]. Further, it is associated with generally worse outcomes as indicated by longer ICU stays and, in some studies, relatively high mortality [1].

Although there are potential causes, such as the military or prisons, where overcrowding is a very important risk factor, it is not so common to observe these cases, especially when dealing with a pathogen that does not rank in the first 10 causes of pneumonia in the United States [7,8]. This case that we present is quite interesting, as this individual did not present with risk factors that could potentially have contributed to it.

In this era, it is not common to observe GAS pneumonia, especially in a developed country where resource-intensive medicine dominates, although cases of GAS pneumonia associated with COVID-19 have been reported, as detailed by Coşkun et al. in their case series, suggesting a possible rise in incidence. On the other side, a time-series analysis of 25 university hospitals, done by Amarsy et al., discussed the rise in GAS invasive infections (not restricted to pneumonia) detected in blood cultures, after the first wave of COVID-19 in 2020, yet this infection rate eventually decreased by 28% due to extreme contingency measures implemented in this hospitals [9,10]. The literature is limited on whether or not there is a rise in GAS pneumonia due to COVID-19.

By reporting more cases of this relatively uncommon cause of pneumonia, we hope to generate increased awareness of a serious, albeit uncommon pathogen. By contributing to the current body of knowledge on these cases and building an understanding of how GAS pneumonia typically presents, appropriate antibiotics and monitoring can be initiated earlier and potentially improve patient outcomes. Further research on this topic could include analyzing many such cases and the treatments employed to determine the optimal antibiotic course. Additionally, during the course of this case, there was concern for TB as the pathogen, but if a sufficient number of cases like this one are identified then GAS could be added to the differential diagnosis of other cases presented with classic TB findings, including hemoptysis and focal pneumonia, as this one did.

## Conclusions

Overall, Streptococcus pyogenes is an uncommon cause of invasive pneumonia, but given its relatively more severe clinical course it is an important area of research. Though the case presented here had a positive outcome, cases in other situations, like with delays in presentation or in high-risk patients, can have worse outcomes, so understanding this pathogen more fully can have important effects on patient survival and other outcomes.
